# Promoters from the itaconate cluster of *Ustilago maydis* are induced by nitrogen depletion

**DOI:** 10.1186/s40694-017-0040-3

**Published:** 2017-11-28

**Authors:** Thiemo Zambanini, Sandra K. Hartmann, Lisa M. Schmitz, Linda Büttner, Hamed Hosseinpour Tehrani, Elena Geiser, Melanie Beudels, Dominik Venc, Georg Wandrey, Jochen Büchs, Markus Schwarzländer, Lars M. Blank, Nick Wierckx

**Affiliations:** 1 Institute of Applied Microbiology – iAMB, Aachen Biology and Biotechnology – ABBt, RWTH Aachen University, Worringerweg 1, Aachen, 52074 Germany; 2 BioSC, c/o Forschungszentrum Jülich, 52425 Jülich, Germany; 3 Department of Biology, Philipps-University Marburg, Karl-von-Frisch-Straße 8, 35032 Marburg, Germany; 4 AVT-Aachener Verfahrenstechnik, Biochemical Engineering, RWTH Aachen University, Forckenbeckstraße 51, 52074 Aachen, Germany; 5 Institute of Crop Science and Resource Conservation (INRES), Rheinische Friedrich-Wilhelms-Universität Bonn, 53113 Bonn, Germany; 6 Institute of Plant Biology and Biotechnology (IBBP), Westfälische Wilhelms-Universität Münster, 48143 Münster, Germany

**Keywords:** *Ustilago maydis*, Itaconate cluster, Promoter characterization, GFP

## Abstract

**Background:**

*Ustilago maydis* is known for its natural potential to produce a broad range of valuable chemicals, such as itaconate, from both industrial carbon waste streams and renewable biomass. Production of itaconate, and many other secondary metabolites, is induced by nitrogen limitation in *U. maydis*. The clustered genes responsible for itaconate production have recently been identified, enabling the development of new expression tools that are compatible with biotechnological processes.

**Results:**

Here we report on the investigation of two of the native promoters, P_*tad1*_ and P_*mtt1*_, from the itaconate cluster of *U.* *maydis* MB215. For both promoters the specific activation upon nitrogen limitation, which is known to be the trigger for itaconate production in *Ustilago*, could be demonstrated by *gfp* expression. The promoters cover a broad range of expression levels, especially when combined with the possibility to create single- and multicopy construct integration events. In addition, these reporter constructs enable a functional characterization of gene induction patterns associated with itaconate production.

**Conclusions:**

The promoters are well suited to induce gene expression in response to nitrogen limitation, coupled to the itaconate production phase, which contributes towards the further improvement of organic acid production with *Ustilago*.

## Background

The family of Ustilaginaceae has sparked great interest as promising industrial production organisms in recent years. The growing biotechnological attention results from their native ability to utilize a range of bio-based substrates [10, 18, 20, 62] and to produce a broad variety of value–added chemicals, such as glycolipids, polyols, and organic acids [12, 19, 33]. Considerable efforts have been undertaken to optimize fermentation and process conditions in order to increase the yield, titer and rate of glycolipids [42], erythritol [28], malate [61], itaconate [16, 41] and 2-hydroxyparaconate [15, 22]. The biochemical pathways and associated gene clusters of several secondary metabolites have been characterized and engineered, including those of cellobiose lipids [51], mannosyl erythritol lipids [23, 27], malate [60], itaconate [17, 59], and 2-hydroxyparaconate [16]. These efforts have accelerated recently largely thanks to a growing suite of efficient genetic engineering tools, including marker recycling through the FLP/FRT system [32], Golden Gate Cloning [53] and Cas9-based genome engineering [48]. Although originally developed for the model organism *U. maydis*, these tools can also be adapted for use in other Ustilaginaceae [59, 60]. However, critical limitations remain that are characteristic for fungal biotechnology, including the availability of suitable promoters. Specific sets of promoters have been developed for the production of proteins and chemicals of high industrial relevance in different organisms, such as *Aspergillus niger* [55, 56], *Escherichia coli* [37, 38], and *Penicillium chrysogenum* [44]. Modern online analysis systems have become an attractive means to investigate promoter properties, enabling high-resolution characterization of the specific time of induction, the induction trigger, or the promoter strength, for different conditions or promoter variants [64]. One particularly important trigger for the production of organic acids and glycolipids in Ustilaginaceae is nitrogen limitation. Such a limitation causes a strict temporal separation of the very different cellular objectives associated with biomass growth and product formation [14, 26, 52]. This characteristic of fungal secondary metabolite production may be exploited for metabolic engineering. Constitutive promoters, such as *P*_*otef*_ and *P*_*oma*_ [13, 46, 49], are mainly active in the growth phase, while inducible promoters, such as *Pcrg1*, *Pnar1* or the tet-system, come with other drawbacks [4, 6, 63]. *Pcrg1* relies on arabinose and is efficiently repressed by glucose and xylose, two of the main carbon sources for industrial production processes [4]. *Pnar1* is induced by nitrate and repressed by ammonium [6] and is therefore not suitable for nitrogen-limited production. Other inducible promoters are associated with specific phases of the *Ustilago* life cycle, making them equally problematic for engineering of secondary metabolite production [2]. Hence new promoters, which are specifically activated by nitrogen limitation hold much promise for metabolic engineering.

Recently, the gene cluster responsible for itaconate production in *U.* *maydis* was identified [17] and its promoters are promising candidates to overcome current limitations. From the different functions of the genes in the cluster, including catalytic, transport and transcriptional regulation activities, it may be deduced that the individual promoters should possess different inherent properties with respect to induction and strength. This was confirmed by qRT–PCR [17]. Two- to ninefold elevated activity for all seven genes in this cluster, except for *rdo1*, was shown during the itaconate production phase compared to non-induced conditions in rich medium [17]. Further, the regulation of most genes in the cluster is strongly dependent on the putative transcriptional regulator *ria1*. By deletion of *ria1*, the transcription level for all genes within the cluster was lowered by up to ninefold and overexpression triggered expression of most cluster genes [17]. Therefore these promoters show much promise for biotechnological usage as expression tools.

Here we investigate a set of promoters from the genes responsible for itaconate production in *U. maydis* that are induced during the non-growing production phase as initiated by nitrogen limitation.

## Methods

### Strains and cultivation conditions

All strains used and constructed within this work are listed in Table 1.

**Table 1:** Strains used and constructed within this work

Name	Genotype	Origin
*Wildtype strains*		
*U. maydis* MB215	Wildtype	DSM 17144
*E. coli* DH5α	Wildtype	DSM 6897
*Recombinant U. maydis strains*		
*U. maydis* MB215 *P*_*otef*_-*gfp* (s)	Single integration of pUMa43-otef-gfp-nos cbx	This work
*U. maydis* MB215 *P*_*otef*_-*gfp* (m)	Multiple integration of pUMa43-otef-gfp-nos cbx	This work
*U. maydis* MB215 *P*_*tad1*_-*gfp* (s)	Single integration of pUMa43-*P*_*tad1*_-gfp cbx	This work
*U. maydis* MB215 *P*_*tad1*_-*gfp* (m)	Multiple integration of pUMa43-*P*_*tad1*_-gfp cbx	This work
*U. maydis* MB215 *P*_*itp1*_-*gfp* (s)	Single integration of pUMa43-*P*_*itp1*_-gfp cbx	This work
*U. maydis* MB215 *P*_*itp1*_-*gfp*(m)	Multiple integration of pUMa43-*P*_*itp1*_-gfp cbx	This work
*U. maydis* MB215 *P*_*adi1*_-*gfp* (s)	Single integration of pUMa43-*P*_*adi1*_-gfp cbx	This work
*U. maydis* MB215 *P*_*adi1*_-*gfp* (m)	Multiple integration of pUMa43-*P*_*adi1*_-gfp cbx	This work
*U. maydis* MB215 *P*_*mtt1*_-*gfp* (s)	Single integration of pUMa43-*P*_*mtt1*_-gfp cbx	This work
*U. maydis* MB215 *P*_*mtt1*_-gfp (m)	Multiple integration of pUMa43-*P*_*mtt1*_-gfp cbx	This work

As host for cloning experiments, *E.* *coli* DH5α (DSM 6897) was used. All *E.* *coli* strains were grown at 37 °C shaking at 200 rpm (shaking diameter 25 mm) in lysogeny broth (LB) medium. For recombinant strains 100 mg L^−1^ ampicillin were added to the medium.

*Ustilago* *maydis* cultures were cultivated in YEPS light medium containing 20 g L^−1^ D–sucrose, 5 g L^−1^ yeast extract, and 10 g L^−1^ peptone at 30 °C shaking at 200 rpm (shaking diameter 25 mm).

For physiological experiments 96 flat-bottom round-well plates or 48-well flower plates were used as described below.

As cultivation medium MTM was used containing 50 g L^−1^ glucose, 0.2 g L^−1^ MgSO_4_ · 7 H_2_O, 0.01 g L^−1^ FeSO_4_ · 7 H_2_O, 0.5 g L^−1^ KH_2_PO_4_, 1 mL L^−1^ vitamin solution, 10 mL L^−1^ trace element solution, 19.5 g L^−1^ 2-(N-morpholino)ethanesulfonic acid (MES) as buffer, and differing NH_4_Cl concentrations. MES buffer was used as stock solution with pH adjusted to 6.5 with NaOH. The vitamin solution contained (per liter) 0.05 g D-biotin, 1 g D-calcium panthotenate, 1 g nicotinic acid, 25 g myo-inositol, 1 g thiamine hydrochloride, 1 g pyridoxol hydrochloride, and 0.2 g para–aminobenzoic acid. The trace element solution contained (per liter) 1.5 g EDTA, 0.45 g ZnSO_4_ · 7 H_2_O, 0.10 g MnCl_2_ · 4 H_2_O, 0.03 g CoCl_2_ · 6 H_2_O, 0.03 g CuSO_4_ · 5 H_2_O, 0.04 g Na_2_MoO_4_ · 2 H_2_O, 0.45 g CaCl_2_ · 2 H_2_O, 0.3 g FeSO_4_ · 7 H_2_O, 0.10 g H_3_BO_3_, and 0.01 g KI [19]. For phosphate limitation 3.2 g L^−1^ NH_4_Cl and 0.1 g L^−1^ KH_2_PO_4_ were used.

Precultures for analytical experiments were cultivated for 24 h in 500 mL shake flasks without baffles with 50 mL MTM containing 4 g L^−1^ NH_4_Cl, to ensure that no nitrogen limitation occurs prior to inoculation of the main culture.

### Analytical methods

All experiments were performed in triplicates, unless stated otherwise. The arithmetic mean of the biological replicates is shown. Error bars and ± values indicate standard error of the mean.

HPLC analysis was performed as described previously [62]. Centrifuged samples (13,000*g*, 5 min) were filtered through cellulose acetate filters (diameter 0.2 µm, VWR, Germany) and subsequently diluted 1:10 with distilled water. For analysis a Dionex Ultimate 3000 HPLC (Dionex, USA) with an Organic Acid Resin column (CS-Chromatographie, Germany) kept at 75 °C, with a constant flow rate of 0.8 ml min^−1^ of 5 mM sulfuric acid as eluent was used. For detection, a Shodex RI101 detector at 35 °C and a variable wavelength UV detector (Dionex, USA) at 210 nm were used.

Ammonium concentration was determined by a colorimetric assay according to Willis [57]. For this 50 µL of the sample (maximal 50 mg L^−1−^ NH_4_^+^) were mixed with 1 mL reagent solution and afterwards with 0.25 mL hypochlorite solution. The mixture was incubated for at least 12 min at room temperature before measuring the absorbance at 685 nm. The reagent solution contained 32 g sodium salicylate (anhydrous), 40 g trisodium phosphate (TSP) and 0.5 g sodium nitrosylpentacyanoferrate(III) (sodium nitroprusside) dissolved in 1 L of water. For the hypochlorite solution, 50 mL of commercially available bleach (Clorox) containing 5–5.25% sodium hypochlorite were diluted with water to 1 L with a final concentration of ~ 0.25% hypochlorite.

Initial promoter characterization experiments were performed in 96-well plates using a BioLector (m2p-labs, Baesweiler, Germany) at 30 °C shaking at 1000 rpm (shaking diameter: 3 mm). GFP fluorescence intensity was determined at 488/520 nm (excitation/emission) with the gain set to 70. For biomass determination backscatter intensity in the far red range 620/620 nm with the gain set to 10 was used. Specific promoter activities are expressed as GFP fluorescence over biomass backscatter. Since both these signals are measured in arbitrary units, this specific activity is expressed without dimension. Values are normalized (point-to-point) for the arithmetic mean of the corresponding wildtype. Induction ratios were determined by dividing the maximum specific promoter activity by the average activity from 2–10 h.

Induction profiles of *U. maydis* MB215 *P*_*tad1*_-*gfp* under different cultivation conditions were analyzed in 48-well flower plates (M2P-labs, Baesweiler, Germany) with an in-house constructed screening system based on the established BioLector setup [45, 54] with a fluorospectrometer, which features excitation and emission monochromators for free wavelength selection in the UV/Vis range (Fluoromax-4, HORIBA Jobin–Yvon). GFP fluorescence intensity was determined at 495/507 nm (bandpass: 8 nm, integration time: 600 ms) and biomass was measured via backscatter intensity at 650/650 nm (bandpass: 4 nm, integration time: 1200 ms).

### Cloning procedures

The genome of *U.* *maydis* 521 was taken as reference sequence [29].

**Fig. 1:**
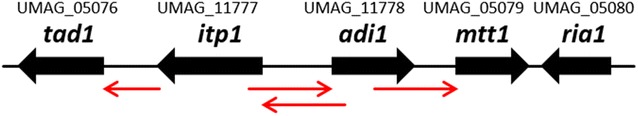
Clustered itaconate genes in *U.* *maydis* including *trans*-aconitate decarboxylase (*tad1*), a major facilitator superfamily transporter (*itp1*), an aconitate–Δ–isomerase (*adi1*), a mitochondrial tricarboxylate transporter (*mtt1*), and a transcriptional regulator (*ria1*) [17]. Red arrows indicate the promoter regions investigated in this study

Plasmids *P*_*tad1*_-*gfp*, *P*_*itp1*_-*gfp*, *P*_*adi1*_-*gfp*, and *P*_*mtt1*_-*gfp* were cloned by amplifying the promoter regions of genes *UMAG_05076* (*tad1*), *UMAG_05077* (*itp1*), *UMAG_05078* (*adi1*), and *UMAG_05079* (*mtt1*) with the oligonucleotide primers listed in Table 2 and exchanging *P*_*otef*_ on the vector pUMa43 [34]. For *UMAG_05077* (*itp1*), *UMAG_05078* (*adi1*), and *UMAG_05079* (*mtt1*) 2000 bp in front of the respective start codon were used and for *UMAG_05076* (*tad1*) 1500 bp were used (Fig. 1).

**Table 2:** Oligonucleotide primers with restriction sites

Plasmid	Primers/specifications	Restriction enzymes
pUMa43	*P*_*otef*_-*gfp*-T_nos_; ori ColE1; ampR; *U. maydis ip* ^*R*^-locus	
*P*_*tad1*_-*gfp*	fwd: CTCGGTACCTTCGACTTGGTGGATACTGCGGCTGTTG rev: GTCGGTCTTGACTCGACCCAGCTCACCGGATCCGTA	*KpnI*/*BamHI*
*P*_*itp1*_-*gfp*	fwd: TCGAAATTCGAGCTCGGTACCGACCTGACAGAAGAGATAG rev: AGCTCCTCGCCCTTGCTCACCATGGTTCGACTTGGTGGATACTG	*KpnI*/*NcoI*
*P*_*adi1*_-*gfp*	fwd: TCGAAATTCGAGCTCGGTACCTCAGCCGATAGGTTTCAC rev: AGCTCCTCGCCCTTGCTCACCATGGTGAGCTGGGTCGAGTC	*KpnI*/*NcoI*
*P*_*mtt1*_-*gfp*	fwd: TCGAAATTCGAGCTCGGTACCGTTTACCGCACGCTGTA rev: AGCTCCTCGCCCTTGCTCACCATGGTGGATGACGAATCTCAAG	*KpnI*/*NcoI*

The resulting plasmids were linearized with the restriction enzyme *SspI* prior to *U. maydis* transformation and constructs were integrated into the *ip*-locus of *U.* *maydis* strain MB215 by homologous recombination [40]. Transformation of *U. maydis* was performed using standard protocols [47]. For selection of transformants, PDA plates with 2 μg ml^−1^ carboxin were used. Correct integration of constructs and number of integration events was verified by Southern blot analysis using enzyme *EcoRV* for genome restriction and the Cbx-cassette from vector pMF1–C [5] as a probe for the detection of specific DNA fragments.

## Results and discussion

### Expression strengths of promoters from the itaconate cluster of *U.* *maydis*

The recently characterized genes encoding core catalytic enzymes and transporters, which are required for itaconate production (*tad1*, *itp1*, *adi1*, *mtt1*) were chosen as primary targets for the investigation of promoter activities. From previous studies it was known that their native activity is strongly influenced by the activity of the transcriptional regulator Ria1 which is itself also encoded in the itaconate gene cluster [17].

For analysis of induction conditions and promoter strength we fused each of the four promoters to *gfp*. GFP has been shown to be a suitable tool for expression analysis in several organisms including *Escherichia* *coli* [11, 43], *Saccharomyces* *cerevisiae* [1] and *Pseudomonas* *putida* [64]. Also in *U.* *maydis* GFP was used before to investigate host–pathogen interactions [49], to identify and localize proteins, for example a motor protein [39], and to investigate promoter activities [3, 49].

**Fig. 2:**
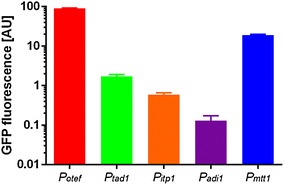
Fluorescence signals for *U.* *maydis* single integration mutants cultivated in MTM containing 0.8 g L^−1^ NH_4_Cl and 50 g L^−1^ glucose expressing *gfp* under the control of four promoters from the itaconate gene cluster. GFP fluorescence intensities (normalized against the wildtype) are shown for single integration mutants under control of *P*_*otef*_ (red), *P*_*tad1*_ (green), *P*_*itp1*_ (orange), *P*_*adi1*_ (purple), and *P*_*mtt1*_ (blue) after 24 h of cultivation. Error bars indicate standard error of the mean (*n* = 3)

The plasmids containing the promoter-*gfp* fusions were integrated into the *ip*-locus of *U.* *maydis* MB215 [9, 31]. The resulting mutants were screened for *ip*–locus and single (s) or multiple (m) integration events by PCR and Southern Blotting. This targeted in-locus integration avoids undesired polar effects, such as gene disruption, and ensures optimal comparability between different strains by avoiding locus- and copy number-dependent expression differences [49]. For all promoters, one clone with single construct integration was chosen for further characterization (Fig. 2).

Fluorescence increased over time for all strains (data not shown), however, with great differences in the absolute intensity range. While *P*_*itp1*_ and *P*_*adi1*_ showed very low fluorescence intensities after 24 h of cultivation, about two-to-three orders of magnitude below that of the strong *P*_*otef*_, *P*_*tad1*_ showed 48-fold and *P*_*mtt1*_ fivefold lower intensities. These data indicate pronounced differences of promoter activity within the itaconate cluster from *U.* *maydis*, with *P*_*adi1*_ and *P*_*itp1*_ as relatively weak promoters and *P*_*mtt1*_ as a strong promoter. The differences are in line with the physiological roles of the regulated genes. The mitochondrial transporter Mtt1 is the limiting step for itaconate biosynthesis in *U.* *maydis* [17], requiring relatively high expression. In contrast, high expression of Adi1 would result in a surplus of *trans*–aconitate, which is a potent inhibitor for vital metabolic reactions, such as the conversion of citrate to isocitrate by aconitase in the TCA–cycle [21]. The *in*-*trans* expression of *P*_*adi1*_ and *P*_*itp1*_ outside of their original genomic context might influence their activity, since the orientation in the genome suggests a possible bidirectional promoter (Fig. 1), which might be influenced by additional upstream elements in their native context. Based on those results, *P*_*tad1*_ and *P*_*mtt1*_ were chosen as the two strongest promoters for further characterization and for the development as expression tools.

### Activation of the *P*_*tad1*_ and *P*_*mtt1*_ occurs in response to nitrogen limitation

**Fig. 3:**
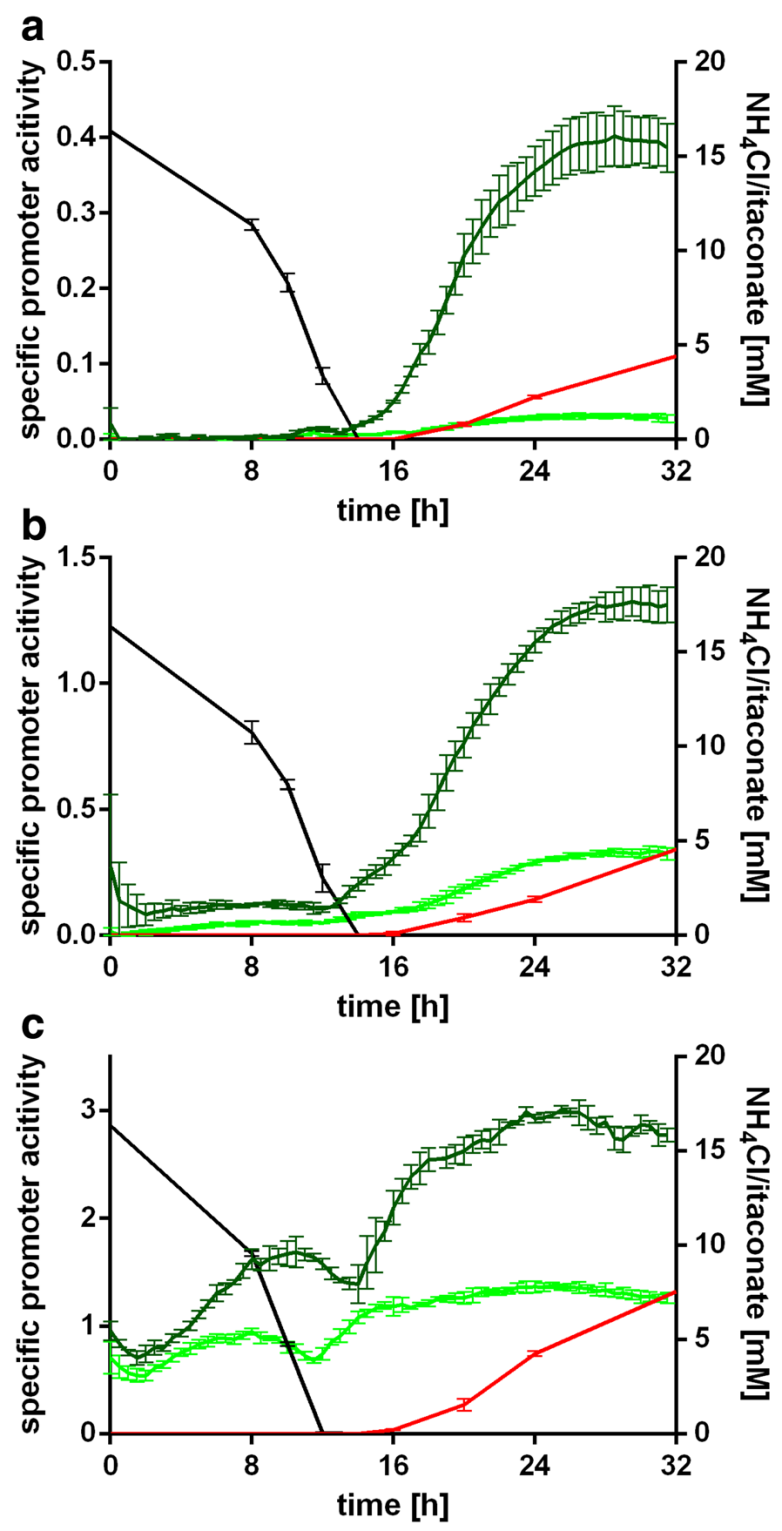
Induction profiles of *gfp* under control of *P*_*tad1*_ (**a**), *P*_*mtt1*_ (**b**), and *P*_*otef*_ (**c**). Itaconate concentration (red) and NH_4_Cl concentration (black) correlated to the specific promoter activity (GFP over biomass, normalized against the WT) for *U. maydis* strains containing single (light green) and multiple (dark green) insertions. Error bars indicate standard error of the mean (*n* = 3)

For expression of genes coupled to itaconate production the induction time can be critical, depending on the expressed product, since some products are toxic during the growth phase, and the production of itaconate poses a drain on the primary metabolite *cis*-aconitate. Consequently we investigated the induction time and conditions (Fig. 3) for *P*_*tad1*_ and *P*_*mtt1*_ using *P*_*otef*_ as a control. Additionally, we investigated the differences resulting from clones with single construct integration compared to multiple integration, hypothesizing that multiple insertions may allow for a wider range of activities. It has to be noted, however, that the copy number for multiple integration events could not be quantified.

Promoter activities are expressed as GFP fluorescence intensity over biomass (measured as scattered light). The activity of both *P*_*tad1*_ and *P*_*mtt1*_ increased strongly after 14 h of cultivation, corresponding to the time point where ammonium was depleted from the culture medium (Fig. 3). Production of itaconate started approximately two hours later. This correlates well with the known induction of itaconate production for *U.* *maydis* [14]. In contrast, the strong constitutive *P*_*otef*_ promoter gave a high and relatively stable signal during the growth- and production phases. The low fluorescence intensity of the *P*_*tad1*_ construct during the initial growth phase hints at a weak, but tightly controlled activity with an induction ratio of 72 (single integration) and 122 (multiple integrations). *P*_*mtt1*_ in contrast is a strong, but leaky promoter, with a basal activity during the growth phase and a lower induction ratio of 13 (single integration) and 11 (multiple integrations). The basal activity of *P*_*mtt1*_ in the growth phase may result from the generally high promoter activity, or from an activation due to expression out of the original chromosomal context.

We next investigated the impact of multiple integration on the expression activity. With values of 0.03 (*P*_*tad1*_, single, 23–32 h), 0.40 (*P*_*tad1*_, multi, 28–29 h), 0.33 (*P*_*mtt1*_, single, 27–32 h), 1.32 (*P*_*mtt1*_, multi, 29–32 h), 1.38 (*P*_*otef*_, single, 25–26 h), and 3.00 (*P*_*otef*_, multi, 25–26 h), the maximum expression level increased by about twofold (*P*_*otef*_), fourfold (*P*_*mtt1*_), and 13-fold (*P*_*tad1*_) for multiple construct integrations compared to single integration. Taking into consideration the promoter activities of single-copy *P*_*tad1*_ and *P*_*mtt1*_ constructs, and multiple integration, expression levels covering a nearly 50-fold dynamic range with high resolution appears realistic. This value may even be increased by selecting higher integration numbers for *P*_*mtt1*_.

### The impact of growth limiting nutrients on *P*_*tad1*_

**Fig. 4:**
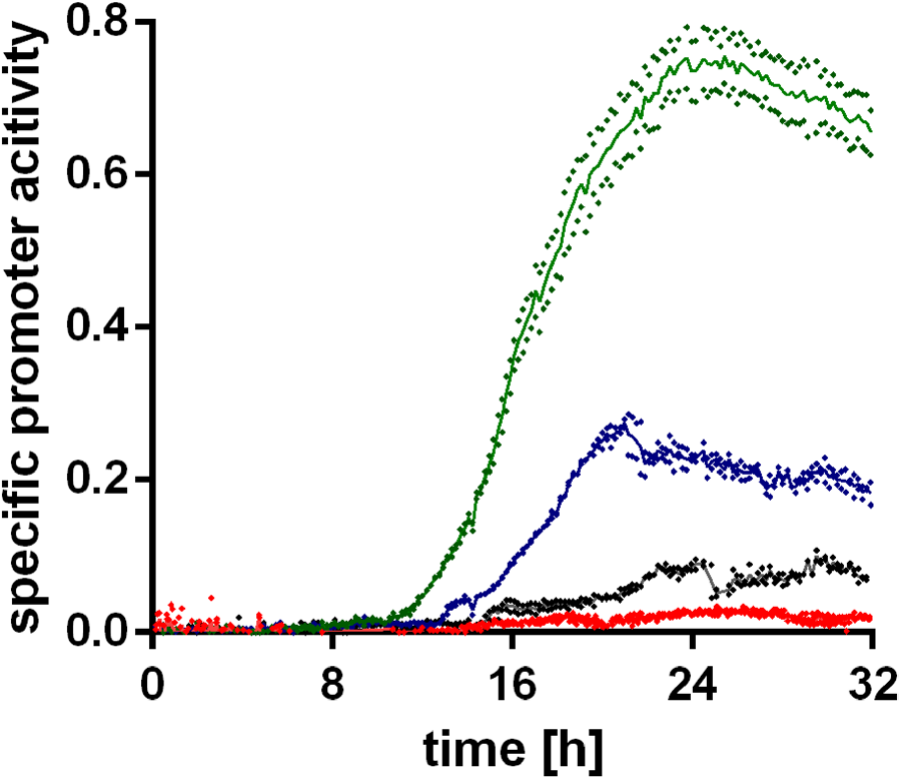
Impact of growth limiting nutrients on *P*_*tad1*_ activity. Specific promoter activity (GFP over biomass, normalized against the wildtype) for *U.* *maydis P*_*tad1*-_ *gfp* cultivated in MTM containing 0.5 g L^−1^ KH_2_PO_4_ with 0.8 g L^−1^ (green), 1.6 g L^−1^ (blue), and 3.2 g L^−1^ (black) NH_4_Cl, and 0.1 g L^−1^ KH_2_PO_4_ with 3.2 g L^−1^ NH_4_Cl (red). Single values are shown for two biological replicates (*n* = 2; diamonds) and the mean value (line)

Itaconate production by *U. maydis* is generally induced upon nitrogen limitation. However, the amount of nitrogen source as a growth-limiting nutrient has a strong impact on the efficiency of itaconate production [16, 41]. In other itaconate producers, such as *Aspergillus terreus*, phosphate-limiting media are generally used [36, 58], although a P-limitation is not strictly needed for efficient induction [25, 35]. In order to further investigate the effect of different growth limitations on the activation of *P*_*tad1*_, we cultivated *U.* *maydis* *P*_*tad1*_-*gfp* with different combinations of NH_4_Cl and KH_2_PO_4_. Keeping the initial concentration of KH_2_PO_4_ at 0.5 g L^−1^ we increased the initial NH_4_Cl concentrations from 0.8 to 1.6 and 3.2 g L^−1^, resulting in higher biomass concentrations and nitrogen limitation at a later time-point during cultivation. To ensure a phosphate-limited culture, we lowered the initial concentration of KH_2_PO_4_ to 0.1 g L^−1^ combined with an initial NH_4_Cl concentration of 3.2 g L^−1^ (Fig. 4).

Higher NH_4_Cl concentrations resulted in a delay of *P*_*tad1*_ induction, correlated with a later depletion of nitrogen. However, specific *P*_*tad1*_ promoter activity was about 2.5-fold (1.6 g L^−1^) and 7.5-fold (3.2 g L^−1^) lower for the cultures containing more NH_4_Cl, compared to the culture containing 0.8 g L^−1^ NH_4_Cl, and for the phosphate-limited culture the specific promoter activity was negligible (Fig. 4). These data further support the role of nitrogen limitation as major trigger for the production of itaconate, as opposed to general growth limitation. They also explain previous observations, where the use of 4 and 0.8 g L^−1^ NH_4_Cl resulted in similar volumetric production rates [16], even though much more biomass was formed at 4 g L^−1^ NH_4_Cl. Apparently, lower nitrogen concentrations result in a stronger induction of itaconate production genes, leading to more efficient production. This may be related to pH in the employed system of batch cultures with a soluble 100 mM MES buffer starting at pH 6.5. Higher nitrogen concentrations may lead to a stronger pH drop during growth, and itaconate formation might be triggered at a sub-optimal pH level with preference over other secondary metabolites. Indeed, *U. maydis* favors the production of glycolipids over organic acids at a low pH [24, 27, 50], and the regulatory interplay between different secondary metabolites is complex [7, 8, 30]. A strong relationship between pH and induction of itaconate production has also been observed for *A. terreus* [35].

In summary, we were able to show that the activation of the itaconate cluster in *U. maydis* is induced specifically in response to nitrogen limitation, and the level of induction was strongly dependent on the initial nitrogen concentration. Although the depletion of nitrogen triggers the activation, different other factors, such as pH and the concentration of other nutrients, can be optimized for fine tuning of the promoters activities.

## Conclusions

The potential of Ustilaginaceae as production organisms for different industrially-relevant compounds has been convincingly demonstrated in several instances. The presented investigation of the promoters from *Ustilago*’s itaconate cluster provides a new set of genetic tools that will enable heterologous gene expression under nitrogen limitation. Activity of these promoters is clearly coupled to the production phase, with a broad range of activities that reach up to the level of the commonly used *P*_*otef*_. The investigation of these promoters opens new doors for future metabolic engineering strategies. These strategies aim for an improved match with the different cellular objectives during growth- and production phases compared to *P*_*otef*_ and *P*_*oma*_, which are mainly active during growth phase or e.g. *Pcrg1* and *Pnar1*, which are activated or repressed under conditions incompatible with *Ustilago’*s production phase. In addition, the GFP fusions enable further detailed investigations into the mechanism of induction of secondary metabolite production in *U. maydis*, and specifically give further insight into the regulation of the itaconate cluster of *Ustilago* and of the itaconate production pathway.
